# Sex-related upregulation of bone morphogenetic protein signaling inhibits adult neurogenesis in APP^NL−G−F^ alzheimer’s disease model mice

**DOI:** 10.1186/s13293-025-00799-0

**Published:** 2025-12-12

**Authors:** Xingyu Su, Rina Takayanagi, Hiroki Maeda, Takaomi C. Saido, Toshio Ohshima

**Affiliations:** 1 Department of Life Science and Medical Bioscience, Waseda University, Shinjuku- ku, Tokyo, 162-8480 Japan; 2 Laboratory for Proteolytic Neuroscience, RIKEN Center for Brain Science, 2-1 Hirosawa, Wako-shi Saitama, 351-0198 Japan; 3 Laboratory for Molecular Brain Science, Department of Life Science and Medical Bioscience, Waseda University, 2-2 Wakamatsu-cho, Shinjuku-ku, Tokyo, 162-8480 Japan

## Abstract

**Background:**

Bone morphogenetic proteins (BMPs) have been reported in many studies to be related to adult neurogenesis. Neurogenic impairment is a hallmark of Alzheimer’s disease (AD), while the involvement of BMPs remains unclear.

**Methods:**

AD models were established using APP^NL−G−F^ transgenic mice and C57BL/6 mice subjected to intracerebral injection of Aβ_(25–35)_ peptide. Female APP^NL−G−F^ mice received pharmacological inhibitor treatment, whereas Neuro2a cells were exposed to estrogen stimulation in vitro. Immunofluorescence staining was conducted to evaluate hippocampal neural stem cell proliferation. The hippocampus and cellular pellets were isolated, and quantitative PCR (qPCR) was employed to determine mRNA expression levels.

**Results:**

Our study revealed that APP^NL−G−F^ mice and Aβ-injected mice exhibited impaired neurogenesis in the brain, with a clear sex-dependent difference only in APP mice. Several BMPs were markedly upregulated in the hippocampus of AD model mice, with significantly higher expression in females than in males. BMP inhibitor attenuated neural stem cell proliferation deficits in female APP^NL−G−F^ mice. Estrogen stimulation robustly enhanced BMP6 expression in Neuro2a cells.

**Conclusions:**

Our findings reveal a sex-dependent impairment of neurogenesis in APP^NL−G−F^ mice driven by BMP signaling. Blocking BMP signaling enhances adult neural stem cell proliferation in female APP^NL−G−F^ mice, providing a potential therapeutic target for AD.

**Supplementary Information:**

The online version contains supplementary material available at 10.1186/s13293-025-00799-0.

## Plain Language summary

Apart from age, Alzheimer’s disease (AD) also shows sex differences, with women being more likely to develop the disease than men [1, 2]. Although this difference is well documented, the underlying mechanisms remain unclear. Evidence suggests that elevated BMP6 levels in the brains of AD patients and APP transgenic mice are associated with impaired neurogenesis [3]. In our study, we found that the increase in BMPs expression in APP^NL−G−F^ mice is sex-dependent, with female AD mice showing higher BMPs levels in the hippocampus than males. This elevation was linked to reduced neural stem cell proliferation. Neurogenic impairment was more severe in female APP^NL−G−F^ mice than in males. Importantly, blocking BMPs significantly rescued impaired hippocampal neural stem cell proliferation in these mice. Interestingly, stimulating Neuro2a cells with estrogen markedly increased BMP6 levels. Our findings suggested that BMP signaling contributed to sex-dependent neurogenic impairment in AD, and that targeting BMPs could represent a potential therapeutic strategy for restoring neurogenesis in the disease.

**Supplementary Information:**

The online version contains supplementary material available at 10.1186/s13293-025-00799-0.

## Highlights

1. Expression of BMPs was elevated in the hippocampus of APP^NL-G-F^ mice, with females showing higher levels than males.
2. Aβ_(25–35)_-injected mice exhibited neurogenic impairment and increased expression of BMPs, but no sex differences were observed.
3. BMP inhibitor effectively alleviated neurogenic impairment in female APP^NL-G-F^ mice.
4. Estrogen stimulation increased BMP expression in Neuro2a cells.

**Supplementary Information:**

The online version contains supplementary material available at 10.1186/s13293-025-00799-0.

## Introduction

Alzheimer’s disease (AD) is one of the main types of dementia and as of 2025, the prevalence of AD among individuals aged 65 years and older in the United States reached 7.2 million, exceeding 7 million for the first time. Moreover, women accounted for nearly two-thirds of the affected population [4, 5]. According to a study that estimated the global number of people with AD spectrum disorders, approximately 50 million people worldwide are affected by this disease. This number is expected to double to more than 100 million by 2050 [6]. This trend poses unprecedented challenges to public health systems, family caregivers, and socioeconomic structures [7]. As no cure exists, research into the disease’s pathophysiology of the disease and the testing of new drugs is necessary [8]. In addition to age, sex differences exist in the incidence of Alzheimer’s, with women having a higher incidence rate than men, especially those over 85 years of age [1, 2]. Although sex differences in AD incidence have been widely reported, the underlying mechanisms remain unclear.

Neurogenesis is the process by which neural stem cells generate new neurons in certain areas of the brain [9, 10], which is crucial for learning and memory. Patients with AD exhibit impaired neurogenesis, which may be closely associated with cognitive decline and neurodegeneration [11–13]. Proliferating cell nuclear antigen (PCNA) is a well-established marker of cell proliferation that plays a central role in DNA replication and repair. During neurogenesis, PCNA is highly expressed in neural stem and progenitor cells undergoing cell cycle progression, and is therefore widely used to evaluate proliferative activity within neurogenic regions such as the hippocampal dentate gyrus (DG). Previous studies have reported that in Amyloid Precursor Protein (APP) transgenic mice, such as APP/PS1 and APP23, PCNA expression is either diminished or displays aberrant patterns [14, 15].

Bone morphogenetic proteins (BMPs) and transforming growth factor-beta are cytokines with similar receptors and messengers [16]. BMPs are essential for the patterning, development, and functioning of the central nervous system [17]. One study reported that increased levels of BMP6 in the brains of patients with AD and in APP transgenic mice were accompanied by impaired neurogenesis [3]. Blocking the BMP pathway via intracranial delivery of the antagonist noggin rescued age-related stem cell loss in the brains of aging mice with neurodegeneration [18]. Transcription Factor AP-2 Beta (TFAP2B) is a transcription factor that coordinates the migration and differentiation of neural crest cells [19] and has been shown to potentially play a role in the BMP signaling pathway [20].

In Alzheimer’s disease research, different mouse models exhibit substantial variations in their genetic design, the onset and progression of Aβ and tau pathologies, and associated phenotypes. These factors directly influence BMP signaling and hippocampal neurogenesis. Commonly used transgenic models include APP/PS1, 5xFAD, and tau-focused models such as PS19.

One study showed that in the 5xFAD model, the BMP signaling pathway was perturbed in AD-like states, suggesting that BMP signaling may be a key pathway in Aβ-driven inhibition of neurogenesis [21]. In APPswe/PS1ΔE9 mice, pharmacological or genetic blockade of BMP signaling using Noggin or other related antagonists has been shown to partially rescue hippocampal neurogenesis [22]. Futhermore, another study demonstrated that BMP9 reduced the amyloid burden and improved cholinergic and cognitive phenotypes in APP/PS1 [23]. These mouse models overexpress mutant human APP, leading to rapid and extensive amyloid plaque deposition and making them highly suitable for drug screening and early pathological investigations. However, APP overexpression can result in non-physiological fragment accumulation, potentially confounding measurements of hippocampal neurogenesis.

In contrast, the APP^NL−G−F^ knock-in model expresses a humanized Aβ sequence harboring the Swedish, Iberian (Beyreuther), and Arctic triple mutations at the endogenous APP locus, maintaining physiological expression levels [24, 25]. Soluble Aβ species are detectable early in the APP^NL−G−F^ mice, with extracellular amyloid plaques becoming evident in cortex and hippocampus within the first 4–7 months and escalating thereafter. Subsequent synaptic alterations and gliosis are reported as pathology progresses, and measurable cognitive deficits emerge in many behavioral assays within 6–12 months [26, 27]. This model not only recapitulates robust Aβ aggregation and the age-dependent emergence of cognitive deficits but also avoids artifacts associated with non-physiological APP overexpression. Consequently, when investigating the effects of Aβ and BMP signaling on hippocampal neurogenesis, the knock-in model provides a context that more closely reflects physiological conditions, facilitating the translation of animal findings to human pathology. Therefore, using APP^NL−G−F^ as a model to study the effects of BMP on hippocampal neurogenesis in AD can reduce the confounding caused by non-physiological APP overexpression, thereby more reliably assessing the biological relevance and clinical translatability of BMP isoforms. Furthermore, recent studies have demonstrated that BMP inhibition in PS19 mice reduces tau pathology and slows cognitive decline [28], further suggesting a key regulatory role for BMP signaling in the development and progression of Alzheimer’s disease. We performed RNA-seq to examine gene expression in the hippocampus of APP^NL−G−F^ mice to investigate sex differences in gene expression and found that *BMP7* was a differentially expressed gene (Takayanagi et al., under review). Although elevated BMP levels have been observed in other mouse models, their sex-dependent regulation remains unexplored.

In the present study, Neuro2a cells were used for in vitro experiments. Neuro2a is a mouse neuroblastoma cell line that can differentiate into neuron-like cells and express neuronal markers such as βIII-tubulin and MAP2 [29]. Previous studies have also demonstrated that Neuro2a cells expressed estrogen receptors that can be activated by exogenous estrogen [30, 31]. These characteristics make Neuro2a cells an ideal in vitro model for investigating neuronal signaling pathways and estrogen-mediated effects.

In this study, our data indicate that sex differences existed in the activation of the BMP signaling pathway, causing neurogenic impairment in AD models, suggesting that blocking the BMP pathway may be a potential therapeutic target for impaired neurogenesis in AD.

## Methods

### Mice

All mouse experiments were performed in accordance with the guidelines of the Institutional Animal Care and Use Committee of Waseda University. APP^NL−G−F^ mice, harboring three familial AD mutations under the control of the endogenous promoter and carrying the humanized Aβ sequence, were generated as previously described [24, 32]. All mutant mice were homozygous for the targeted mutations and were 6 months old at the time of the experiments. Both sexes were included in the study. Age- and sex-matched C57BL/6J mice, designated as WT (Wild-type) mice in this study, served as the control group for the APP^NL−G−F^ knock-in mice. Genotyping to confirm homozygosity was conducted via polymerase chain reaction (PCR) using the following primers: Forward: 5’-CTCCTTGTGGCTGGCGGTCACAC-3’ and reverse: 5’-CTATCGTGGACCGAGAATGGTCATG-3.’

### Quantitative real-time PCR analysis

Total RNA was extracted from the hippocampal tissues using an RNA extraction reagent (Cat No. U0984B, TaKaRa), followed by reverse transcription into cDNA using a commercial kit (Cat No. FSQ-301, TOYOBO), according to the manufacturer’s instructions. qPCR was performed using SYBR Green (Cat No. QPX-201, TOYOBO), 0.2 µM of each primer, and nuclease-free water. β-Actin was employed as an internal control for normalization. Relative gene expression levels were calculated as fold-changes relative to the control group. The primer sequences used were as follows: BMP4: 5’-GCCGAGCCAACACTGTGAGGA-3’ and 5’-GATGCTGCTGAGGTTGAAGAGG-3’; BMP5: 5’-GGCTTACAGCTCTGTGCAGAGA-3’ and 5’-GGATCGAAGAAGTACCTCGCTTG-3’; BMP6: 5’-CTTTCCTCAACGACGCGGACAT-3’ and 5’-CCTCAGGAATCTGGGATAGGTTG-3’; BMP7: 5’-GGAGCGATTTGACAACGAGACC-3’ and 5’-AGTGGTTGCTGGTGGCTGTGAT-3’; DCX: 5’-CTGACTCAGGTAACGACCAAGAC-3’ and 5’-TTCCAGGGCTTGTGGGTGTAGA-3’; TFAP2B: 5’-AGCGGCATGAACCTATTGGACC-3’ and 5’-GGACTGAGCAAAATACCTCGCC-3’; β-Actin: 5’-CTGAGAGGGAAATCGTGCGT-3’ and 5’-CCACAGGATTCCATACCCAAGA-3’.

### Immunofluorescence staining

Antigen retrieval was performed as previously described [33]. Mice were anesthetized with diethyl ether and perfused with 4% paraformaldehyde (PFA) in phosphate-buffered saline (PBS). The brains were removed and postfixed in 4% PFA at 4 °C overnight. After sequential dehydration in 10% and 20% sucrose in PBS, the tissues were embedded in a 2:1 mixture of 20% sucrose and Tissue-Tek optimal cutting temperature compound. Coronal sections of 14 μm thickness were obtained using a cryostat (Microm HM500M, Leica), mounted on MAS-coated glass slides (Cat No. S9441, Matsunami), and stored at − 20 °C until use. For immunostaining, the sections were permeabilized with 0.1% Triton X-100 in PBS. Following three 5 min PBS washes, sections were blocked with 1% BSA in PBS and incubated overnight at 4 °C with anti-PCNA antibody (Cat No. sc-56, Santa Cruz) (1:100). After incubation with Alexa Fluor-conjugated secondary antibodies (Cat No. AB150116, abcam) (1:400) for 1 h and three additional washes. The nuclei were counterstained with Hoechst (Cat No. H341, DOJINDO) (1:1000) for 15 min. Stained sections were examined under a laser fluorescence microscope.

### Intracerebral stereotaxic injection

Intracerebral stereotaxic injection steps were based on previous studies conducted in our laboratory [34]. Aβ_(25–35)_ and Aβ_(35–25)_ peptides were purchased from Peptide Institute Inc. (Japan), dissolved in sterile water at 1 µg/µL, aliquoted, and stored at − 20 °C. For oligomerization, peptides were incubated at 37 °C for 4 d and then stored at − 80 °C. Aβ_(35–25)_ was used as a control. Wild-type (WT) male and female mice (12–14 weeks old) were anesthetized with isoflurane (2–3% induction, 1–2% maintenance) and positioned in a stereotaxic frame (Narishige SR-5 M-HT). A cranial window was created above the injection site under aseptic conditions. Aβ_(25–35)_ or Aβ_(35–25)_ (control) was unilaterally injected into the dentate gyrus at the following coordinates: AP 0 mm, ML + 1.0 mm, DV − 2.0 mm. Infusions of 3 µL were delivered at 1 µL/min using a 5 µL Hamilton syringe with a 10-gauge beveled needle. The needle remained in place for 2 min after injection to promote diffusion. Incisions were sutured, and mice recovered on a 37 °C heating pad before returning to clean housing. Seven days later, the mice were perfused with 4% PFA in PBS or sacrificed for brain collection.

### LDN193189 injection therapy

The injection method was based on a previous study [35]. Female APP^NL−G−F^ mice aged 5 months were administered LDN193189 (Cat No. SML0559, Sigma-Aldrich) intraperitoneally at a concentration of 8.25 µM and a dose of 10 mL/kg once daily for 21 consecutive days. The mice in the control group received autoclaved water at 10 mL/kg via the same injection procedure. After completion of the injection period, the mice were returned to the animal facility and maintained under normal conditions for one week before being sacrificed for brain tissue fixation and subsequent experiments.

### Cell culture and treatment

Neuro2a cells were obtained from the American Type Culture Collection. The cells were cultured in DMEM (Cat No. 10564011, Thermo Fisher Scientific) supplemented with 10% fetal bovine serum (Cat No. FB1285/500, Biosera) and 1% penicillin-streptomycin (Cat No. 0936734, Nacalai Tesque). All experiments were performed within the first 20 passages following acquisition from the American Type Culture Collection. For treatments, Neuro2a cells were exposed to 0.1 µM 17β-estradiol (E2) (Cat No. E888000, Wako) or sterile water for 24 h and 48 h, respectively. Cells were harvested by trypsinization, followed by centrifugation to remove the supernatant. The resulting cell pellet was washed with PBS, centrifuged again to discard the supernatant, and used in downstream assays.

### Statistical analysis

Statistical analyses were performed according to previously reported procedures [36]. Data are presented as mean ± SEM. Group differences were analyzed using an unpaired, two-tailed Student’s t-test for two-group comparisons, or one-way ANOVA followed by Tukey’s post-hoc test for multiple-group comparisons. Pearson’s correlation analysis was performed to calculate the correlation coefficient (r) and corresponding *p*-value, in order to evaluate the strength and significance of the linear associations. Statistical significance was set at *p* < 0.05 for all analyses, with results indicated by asterisks *p < 0.05, **p < 0.01, ***p < 0.001, ****p < 0.0001. Statistical analyses were performed using GraphPad Prism software version 8.3.0.

## Results

### BMPs showed sex differences in APP^NL−G−F^ mice

**Fig. 1:**
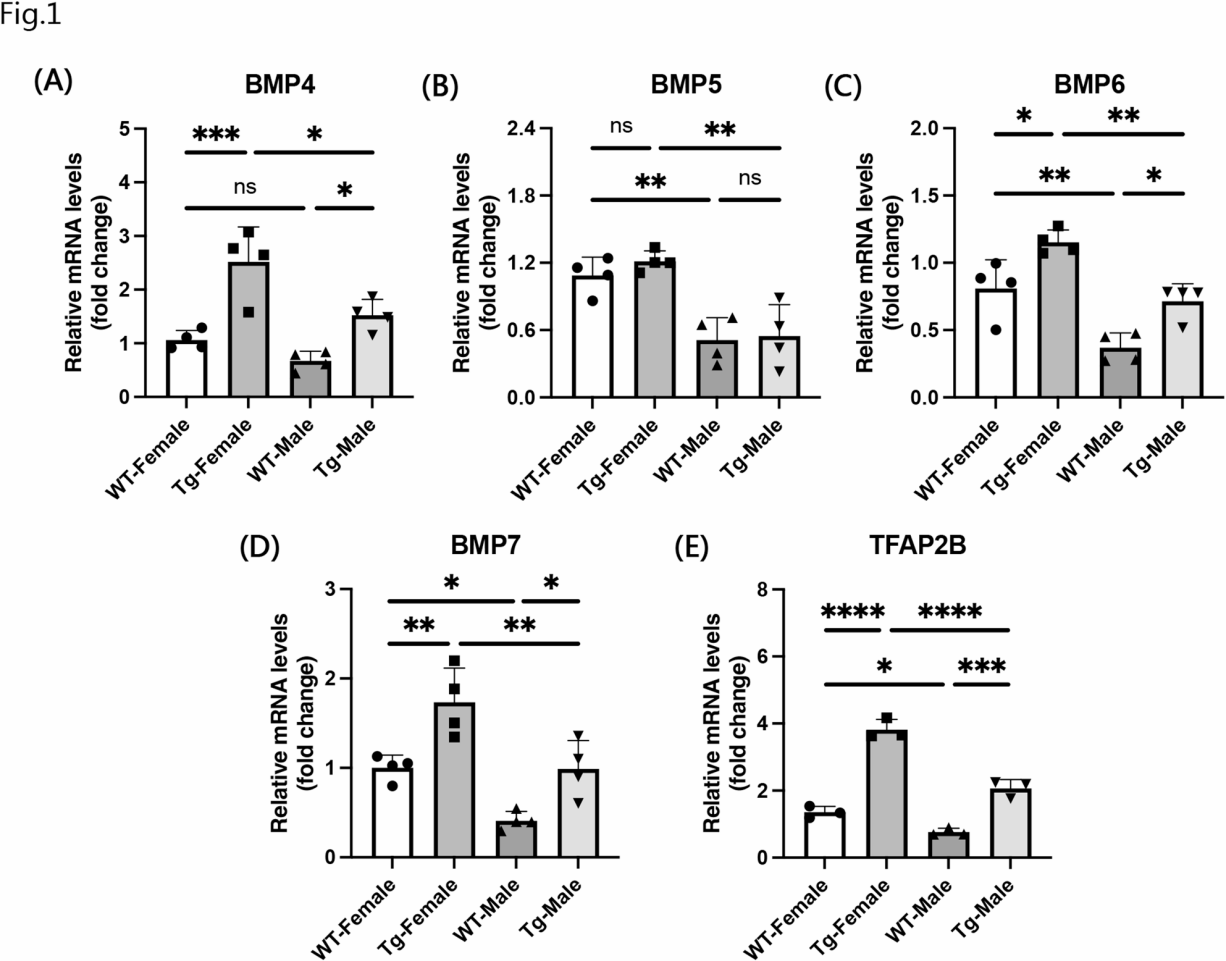
mRNA expression of BMPs and TFAP2B in the hippocampus of 6-month-old WT and APP^NL−G−F^ mice. **A**–**E** The mRNA levels of BMP4 (**A**), BMP5 (**B**), BMP6 (**C**), BMP7 (**D**), and TFAP2B (**E**) in the hippocampus were assayed by qRT-PCR. Data are presented as mean ± SEM; *n* = 3–4 per group. *p < 0.05; **p < 0.01; ***p < 0.001; ****p < 0.0001

A previous study showed that the expression of BMP6 was significantly increased in both patients with AD and APP transgenic mice [3], suggesting a correlation between BMPs and AD pathogenesis. In our previous study (Takayanagi et al., under review), we found that BMP7 was highly expressed in female APP^NL−G−F^ mice. To further substantiate this observation, we conducted qRT-PCR to assess the expression levels of BMP4, BMP5, BMP6, BMP7, and TFAP2B in the hippocampi of 6-month-old APP^NL−G−F^ and WT mice. We also evaluated the expression of doublecortin (DCX), a well-established marker of neuronal differentiation [37, 38]. qPCR results revealed that BMP4 (Fig. 1A), BMP6 (Fig. 1C), BMP7 (Fig. 1D), and TFAP2B (Fig. 1E) expression was significantly upregulated in the hippocampi of APP^NL−G−F^ mice compared to WT controls. However, the BMP5 expression in APP^NL−G−F^ mice was not significantly different from that in WT mice (Fig. 1B). Notably, BMP5, BMP6, BMP7, and TFAP2B expression levels were higher in females than in males in both the WT and APP^NL−G−F^ groups (Fig. 1B–E), supporting the sex differences indicated by the RNA-seq data. A significant sex-dependent increase in BMP4 (Fig. 1A) expression was observed in the APP^NL−G−F^ mice. Additionally, DCX expression was markedly reduced in APP^NL−G−F^ mice, with females exhibiting significantly lower levels than males, suggesting sex-related differences in impaired hippocampal neuronal differentiation associated with APP pathology (Supplementary Fig. 1A). In APP^NL−G−F^ mice, DCX expression was significantly and negatively correlated with BMP4, BMP6, BMP7, and TFAP2B, whereas its correlation with BMP5 was not statistically significant (Supplementary Fig. 2A). These results suggest that the downregulation of DCX may be associated with the increased expression of several BMPs family members and TFAP2B, indicating that the activation of BMP signaling could be linked to impaired neurogenesis in AD.

### Adult neural stem cell proliferation impairment in APP^NL−G−F^ mice presented sex differences

**Fig. 2:**
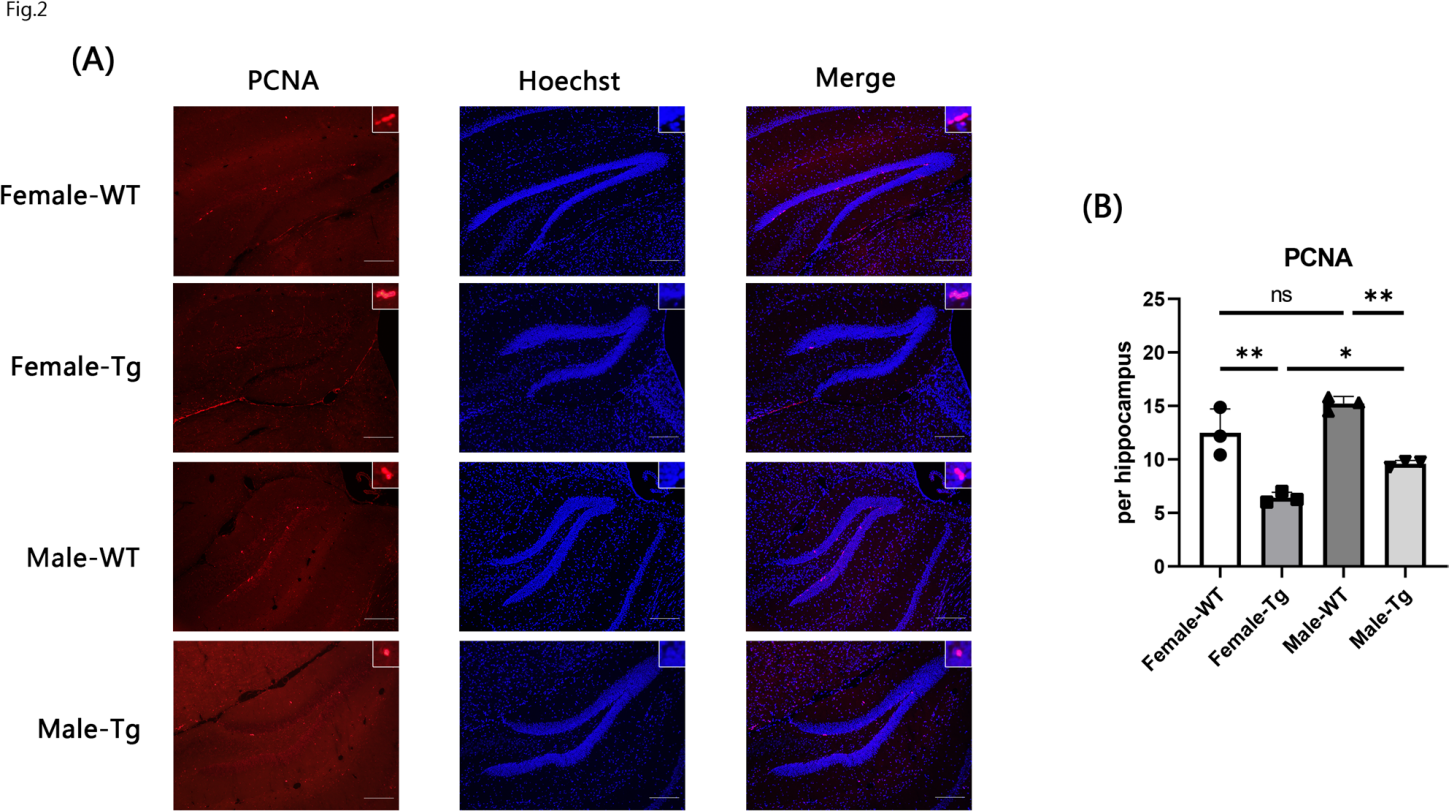
Adult neurogenesis in WT and APP^NL−G−F^ mice. **A** Immunostaining with anti-PCNA antibody (red) and Hoechst (blue) in the dentate gyrus of the hippocampus in 6-month-old WT and APP^NL−G−F^ mice. Scale bar = 100 μm. **B** Quantification of PCNA-positive cells in the DG. Data are presented as mean ± SEM; *n* = 3 per group. *p < 0.05; **p < 0.01; ***p < 0.001

PCNA, a nuclear protein expressed in proliferating cells, served as an indicator of neural stem cell activity and neurogenic capacity [39, 40]. To further investigate hippocampal neural stem cell proliferation in APP^NL−G−F^ mice, we performed immunostaining of frozen brain sections from 6-month-old APP^NL−G−F^ and WT mice using an anti-PCNA antibody (Fig. 2A). Quantitative analysis revealed a significant reduction in the number of PCNA-positive cells in the dentate gyrus of APP^NL−G−F^ mice compared to WT controls (Fig. 2B). Moreover, the number of PCNA-positive cells was significantly lower in female APP^NL−G−F^ mice than in male APP^NL−G−F^ mice. Interestingly, this sex difference in the number of PCNA-positive cell was not observed in the dentate gyrus of WT mice.

### Adult neurogenesis impairment and increased BMPs in Aβ-injected AD model mice

**Fig. 3:**
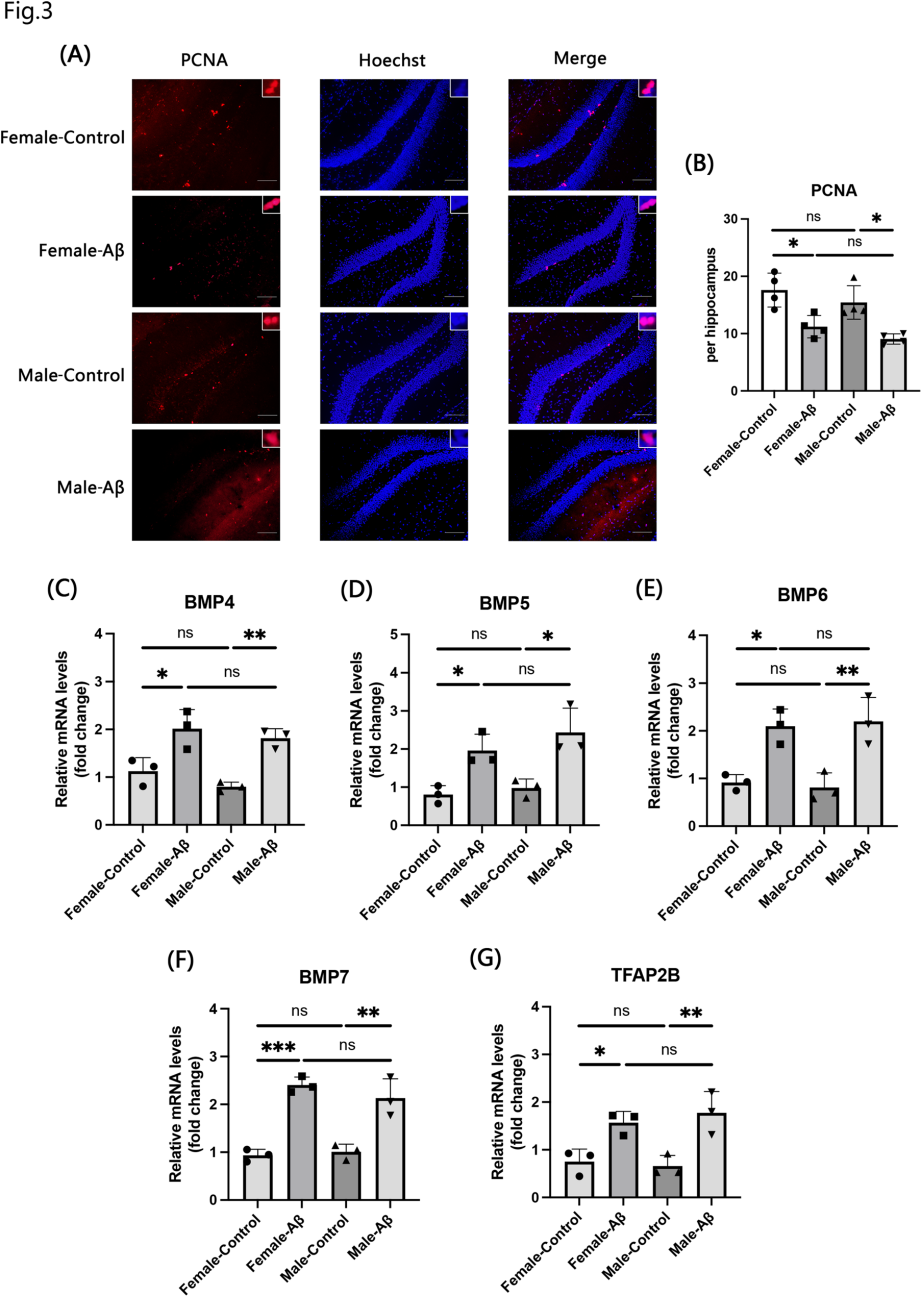
Adult neurogenesis and BMP expression in WT mice with intracerebroventricular Aβ injection. **A** Immunostaining with anti-PCNA antibody (red) and Hoechst (blue) in the dentate gyrus of the hippocampus in 12- to 14-week-old Control mice (injected with Aβ_(35−25)_) and Aβ mice (injected with Aβ_(25−35)_). Scale bar = 100 μm. **B** Quantification of PCNA-positive cells in the DG. **C**-**G** The mRNA levels of BMP4 (**C**), BMP5 (**D**), BMP6 (**E**), BMP7 (**F**), and TFAP2B (**G**) in the hippocampus were assayed by qRT-PCR. Data are presented as mean ± SEM; *n* = 3–4 per group. *p < 0.05; **p < 0.01; ***p < 0.001

The Aβ_(25–35)_ injection model is a widely used acute animal model of AD [41, 42]. Intracerebral injection of synthetic β-amyloid peptide fragments comprising amino acids 25–35 induces AD-like neuropathological features in rodents. Given the altered neurogenesis observed in APP^NL−G−F^ knock-in mice, we aimed to determine whether similar changes occur in the Aβ_(25–35)_ injection model. To this end, brain sections from injected mice aged 12–14 weeks were immunostained with the PCNA antibody (Fig. 3A). In both sexes, the number of PCNA-positive cells was significantly reduced in the Aβ_(25–35)_-injected group compared with that in the control group injected with the reverse sequence peptide Aβ_(35–25)_ (Fig. 3B). However, no significant sex differences in PCNA expression were observed in either the control or Aβ group. We also examined the mRNA expression levels of the BMP signaling components. As shown in Fig. 3C–G, BMP4, BMP5, BMP6, BMP7, and TFAP2B were significantly upregulated in the Aβ group compared with those in the control group. Notably, BMP7 expression showed the most pronounced increase in female mice. However, BMP4, BMP5, BMP6, BMP7, and TFAP2B expression levels did not differ significantly between sexes. As shown in Supplementary Fig. 1B, DCX mRNA expression was significantly decreased in the Aβ group compared with that in the control group. The expression of DCX exhibited a trend consistent with the number of PCNA positive cells (Supplementary Fig. 1B). Similar to that observed in the APP^NL−G−F^ mice, we found that DCX expression was significantly negatively correlated with BMP4, BMP5, BMP6, BMP7, and TFAP2B expression (Supplementary Fig. 2B).

### LDN193189 attenuated adult neural stem cell proliferation impairment in APP^NL−G−F^ mice

**Fig. 4:**
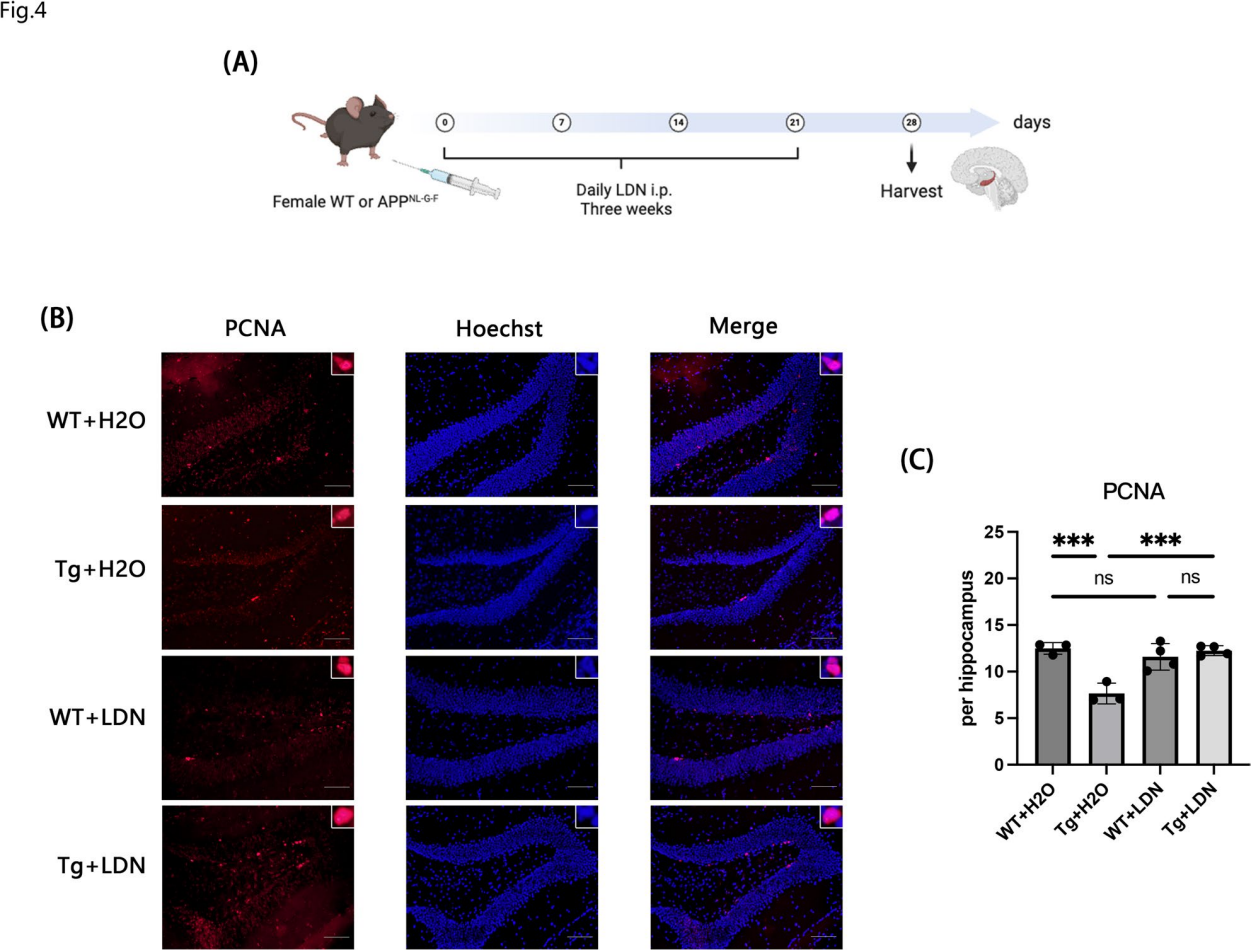
BMP inhibitor promoted neurogenesis in female APP^NL−G−F^ mice. **A** Experimental design for LDN193189 treatment in female APP^NL−G−F^ mice. **B** Immunostaining with anti-PCNA antibody (red) and Hoechst (blue) in the dentate gyrus of the hippocampus in 6-month-old female APP^NL−G−F^ mice intraperitoneally injected with LDN193189 or autoclaved water. Scale bar = 100 μm. **c** Quantification of PCNA-positive cells in the DG. Data are presented as mean ± SEM; *n* = 3–4 per group. **p < 0.01; ***p < 0.001

LDN193189, a BMP signaling inhibitor widely used in biomedical research, inhibits the phosphorylation of the downstream effectors Smad1/5/8 mediated by BMP4, BMP6, BMP7, and related ligands, thereby blocking the activation of the BMP signaling pathway [35, 43]. These results suggested that APP^NL−G−F^ mice exhibited upregulated BMP signaling and impaired neurogenesis. To further investigate the relationship between BMP signaling and neurogenesis, female APP^NL−G−F^ mice were administered daily intraperitoneal injections of 0.01 ml/kg LDN193189, whereas the control group received equivalent volumes of autoclaved water. Treatment was discontinued after 21 days and the brains were fixed 1 week later for immunofluorescence staining (Fig. 4A). The results showed that LDN193189 significantly increased the number of PCNA-positive cells in APP^NL−G−F^ mice (Fig. 4B, C) and restored them to WT levels. These findings confirm that BMP inhibition enhances hippocampal cell proliferation and attenuates neurogenic impairment in female APP^NL−G−F^ mice.

### Estrogen promoted the expression of BMP6 and TFAP2B

**Fig. 5:**
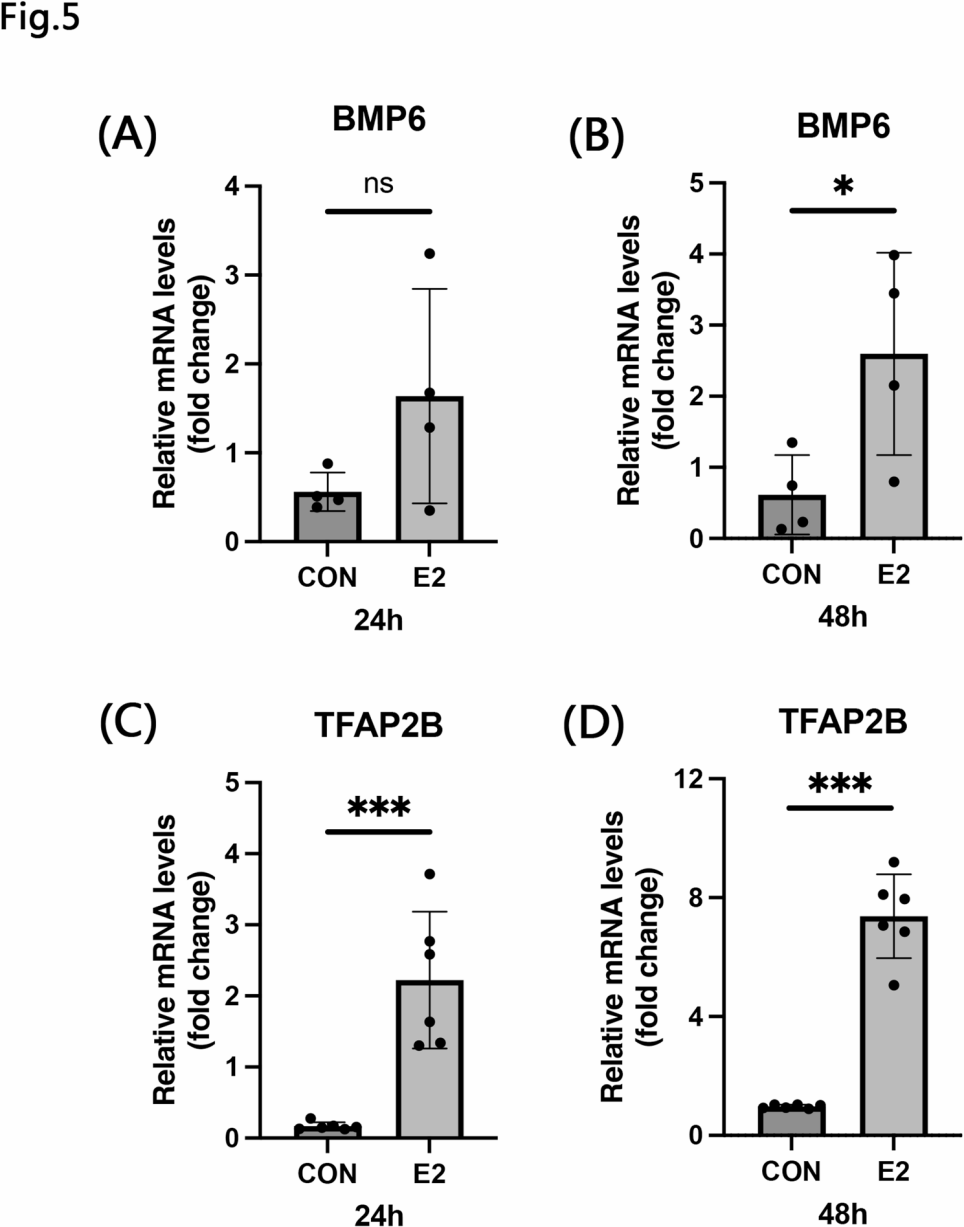
Effects of estradiol on BMP6 and TFAP2B expression in Neuro2a cells. **A**-**D** The mRNA levels of BMP6 (**A** and **B**) and TFAP2B (**C** and **D**) in Neuro2a cells were measured by qRT-PCR following 24 (**A** and **C**) and 48 h (**B** and **D**) estradiol stimulation. Data are presented as mean ± SEM; *n* = 4–6 per group. *p < 0.05; ***p < 0.001

Previous studies have confirmed the presence of estrogen receptors in Neuro2a cells [44, 45]. Moreover, estrogen receptors can transcriptionally regulate the BMP6 promoter [46]. To further investigate the relationship between estrogen and BMPs in vitro, we stimulated Neuro2a cells with E2 and collected samples at 24 and 48 h post-treatment. The results showed that BMP6 mRNA expression was significantly upregulated at 48 h after E2 stimulation (Fig. 5B), whereas TFAP2B expression increased significantly as early as 24 h (Fig. 5C).

## Discussion

Analysis of the Tabula Muris Senis dataset showed that BMP6 and BMP7 expression increased with age in the mouse brain, suggesting that BMP signaling is involved in physiological aging. Moreover, BMP6 displayed sex-dependent expression differences between healthy and Alzheimer’s disease conditions in public scRNA-seq datasets (ADRCSC). Studies have shown that molecular differences exist between male and female brain regions in aging and AD susceptibility and that AD pathogenesis and immune responses also differ by sex [47, 48]. These finding suggest that sex influences AD pathogenesis, disease progression, and potential treatment responses. These findings may explain our observation that BMP expression is elevated in AD patients in a sex-specific manner, which affects AD pathogenesis. Overall, these findings highlight the interplay between aging, sex, and BMP signaling pathways in AD progression.

In Alzheimer’s, BMP family members exert heterogeneous effects on hippocampal neurogenesis. BMP6 is markedly upregulated in the dentate gyrus of AD patients and APP transgenic mice [3, 49], correlating with impaired neurogenesis, suggesting a suppressive role in neuronal generation and maturation. BMP4 similarly inhibits neurogenesis and oligodendrogenesis in AD patient-derived iPSCs, reinforcing its function as an inhibitory regulator [22, 50, 51]. Conversely, BMP9 regulates the survival of early neural progenitors in the adult hippocampus, and its deficiency alters neurogenic dynamics [52]. Exogenous BMP9 administration improves cognition and reduces amyloid pathology in AD mouse models, indicating its proneurogenic and neuroprotective effects [23]. Unlike BMP6, BMP2 and BMP7 show no consistent upregulation in AD [3], and data on BMP5 remain scarce. Our study showed evidence that BMP7 elevation in APP^NL−G−F^ mice was linked to impaired hippocampal neurogenesis, addressing a key gap in the current research. Overall, the existing studies suggest that BMP4 and BMP6 exert inhibitory effects on hippocampal neurogenesis in AD, whereas BMP9 plays a protective regulatory role. These findings highlight the therapeutic potential of BMP signaling and underscore the importance of distinguishing isoform-specific functions in future AD interventions.

This study confirmed that BMP4, BMP6, and BMP7 were significantly upregulated in the APP^NL−G−F^ mice. Concurrently, the hippocampi of these mice exhibited a marked reduction in PCNA-positive cells and DCX mRNA expression (Supplementary Information), indicating pronounced impairment in neurogenesis. To investigate the relationship between neurogenic deficits and BMP signaling upregulation, APP^NL−G−F^ mice were administered the BMP inhibitor, LDN193189, via regular injections to suppress BMP pathway activity. The treatment resulted in a significant alleviation of neural stem cell proliferation impairment in female mice, with recovery levels approaching those observed in WT controls. These findings suggest that BMP signaling constitutes a critical regulatory pathway governing neural stem cell proliferation in this model.

In 6-month-old APP^NL−G−F^ mice, BMP expression was significantly higher in females than in males. To elucidate the mechanism underlying this sex-based difference, we conducted in vitro experiments using Neuro2a cells. Neuro2a cells are of mouse origin, which is consistent with the APP^NL−G−F^ mouse model used in this study. This species consistency helps reduce interspecies variability, making in vitro findings more comparable to in vivo results. Previous studies have employed both APP^NL−G−F^ mice and Neuro2a cells as experimental models [53–55], further supporting the reliability and relevance of this approach. Neuro2a cells are a well-established and easily manipulable cell line that allows for efficient transfection, pharmacological treatment, and mechanistic investigations. Importantly, previous studies [30, 31] have demonstrated the presence of estrogen receptors in Neuro2a cells that can be activated by estrogen, providing a practical basis for estrogen stimulation experiments in this study. Our data showed significant upregulation of BMP6 expression after estradiol stimulation. This result suggests that estrogen signaling may act as an upstream regulator of BMPs, providing testable molecular clues for understanding sex differences in AD. These findings further imply that estrogen could be a potential factor contributing to the elevated BMP transcripts observed in female APP^NL−G−F^ mice.

We also established an acute AD mouse model via intracerebral injection of Aβ_(25–35)_. Although this model exhibited similar BMP signaling upregulation and neurogenic impairment, no significant sex differences were observed. This discrepancy may be attributed to the typically slow and progressive regulatory effects of sex hormones and their associated signaling pathways [56], which require prolonged pathological conditions to manifest sex-specific differences. In contrast, acute models predominantly reflect transient, nonspecific stress or injury responses, potentially obscuring the sex-dependent regulatory mechanisms evident in chronic pathology [57]. Collectively, these data suggest that sex differences in BMP signaling mediated by long-term estrogen action in APP^NL−G−F^ mice may contribute to the higher prevalence of AD observed in females compared with that observed in males. Although sex differences in BMP expression have observed, whether these differences extend to core AD pathology remains uncertain. Previous work has demonstrated that amyloid β deposition and gliosis markers (Iba1 and GFAP) do not significantly differ between male and female APP^NL−G−F^ mice [27]. Therefore, we focused on the BMP-mediated regulation of neurogenesis, while the potential effects on other AD pathologies warrant further investigation.

In qRT-PCR experiments, we observed that the expression pattern of TFAP2B was highly consistent with that of BMP4, exhibiting a positive correlation and upregulation. This result differs from those of previous reports indicating that TFAP2B negatively regulates BMP4 expression by binding to its promoter regions [58]. We speculate that this discrepancy may be because of the abnormal increase in BMP4 in the AD model [59] and that abnormal activation of BMP4 may induce TFAP2B upregulation through feedback mechanisms to maintain neural microenvironment homeostasis or mitigate neuronal damage, reflecting the compensatory enhancement of TFAP2B in a pathological state.

This study has some limitations. Our data showed that estradiol stimulation induced an increase in TFAP2B expression in Neuro2a cells, indicating its potential role in estrogen receptor signaling. Although AD primarily develops in women with low estrogen levels, local alterations in estrogen signaling or compensatory responses may occur during AD-related pathologies. In AD, female may exhibit dysregulation of estrogen signaling pathways or altered receptor sensitivity, which could potentially influence BMP regulation [60, 61]. Therefore, estrogen may represent one factor contribute to the elevated BMP transcripts observed in female APP^NL−G−F^ mice; however, it is unlikely to be the main reason for the sex-biased expression of BMPs in AD. Therefore, the underlying mechanisms must be explored and validated. Previous studies have demonstrated that estrogen exerts both neuroprotective and pro-neurogenic effects [62, 63], which may explain the partial loss of neuroprotection in female patients with AD due to estrogen deficiency. In the present study, estrogen was identified as a factor contributing to BMP6 upregulation. However, there is currently no evidence supporting a direct causal relationship between estrogen-induced BMP6 upregulation and impaired neurogenesis. Whether other upstream or parallel factors influence the relationship between BMPs and neurogenesis requires further investigation.

Adult hippocampal neurogenesis has been well established in rodents; although the number of new neurons declines with age, even low levels of neurogenesis can contribute to memory, cognitive plasticity, and compensation for neural damage [12, 64]. In our previous experiments, no PCNA-positive cells were detected in the hippocampi of 9-month-old APP^NL−G−F^ mice. Nevertheless, exploring the effects of BMP signaling on hippocampal neural stem cell proliferation remains scientifically meaningful [65, 66]. Variations in neurogenesis findings across AD patients and mouse models likely reflect differences in the animal models, age, brain region, markers, and disease stages. While PCNA is a useful indicator of proliferation, it may be influenced by the cell cycle state and AD pathology, and thus may not fully capture the entire neurogenesis process. Our results provide preliminary evidence that BMP signaling regulates neural stem cell proliferation, but further studies using multiple markers and time points are needed to clarify its role in neuronal differentiation and maturation. Lastly, BMP4 expression exhibited sex differences only in APP^NL−G−F^ mice. To date, no study has investigated whether age affects sex-based differences in BMP expression. We speculate that age may be a major factor contributing to the sex differences in BMP5, BMP6, BMP7, and TFAP2B expression in AD. Our results suggest that the abnormal elevation of BMP4 is a major contributor to the pathogenesis of AD in females.

Several autopsy studies have shown that adult hippocampal neurogenesis is impaired in patients with AD [67–69]. A landmark study found that the number of DCX-positive immature neurons in the dentate gyrus of AD patients was significantly lower than that in healthy controls [70]. Subsequent studies showed that hippocampal neurogenesis can continue into late life but is significantly reduced in individuals with AD or severe cognitive impairment, with multiple cohort studies reporting reduced immature neuronal markers and proliferation indices [69, 71]. Reviews and recent single-cell transcriptome analyses also support the loss or transcriptional dysregulation of adult neurogenesis populations in AD [68, 72]. We observed reduced DCX expression and fewer PCNA-positive cells in APP^NL−G−F^ and Aβ-injected mouse models, which was consistent with findings from studies of AD patients. BMP signaling has been reported to be enhanced in the brains of AD patients, particularly in the hippocampus and cortex [3], and we also observed increased BMP signaling in our mouse experiments. This suggests that the upregulation of BMP signaling could be a potential mechanism underlying impaired neurogenesis in human AD. In mouse models, inhibition of BMP signaling restored adult neural stem cell proliferation, indicating its role in neurogenesis. Our findings suggest that targeting BMP signaling is a potential therapeutic strategy for AD.

## Supplementary Information

Supplementary Material 1.

## Data Availability

The data that support the findings of this study are available from the corresponding author upon request.
