# Supplementary material for: Sex-related upregulation of bone morphogenetic protein signaling inhibits adult neurogenesis in APPNL−G−F alzheimer’s disease model mice
Source: Biol Sex Differ. 2025 Dec 12;16:103. doi: 10.1186/s13293-025-00799-0 (PMC12699841; doi:10.1186/s13293-025-00799-0)
Supplement: Supplementary file 1 — Supplementary Material 1. [file 13293_2025_799_MOESM1_ESM.docx]

**Supplemental Information**

**Sex-related enhancement of bone morphogenetic protein signaling inhibits adult neurogenesis in mouse models of Alzheimer's disease**

**Xingyu Su^1^, Rina Takayanagi^1^, Hiroki Maeda^1^, Takaomi C. Saido^2^, Toshio Ohshima^1,3^**


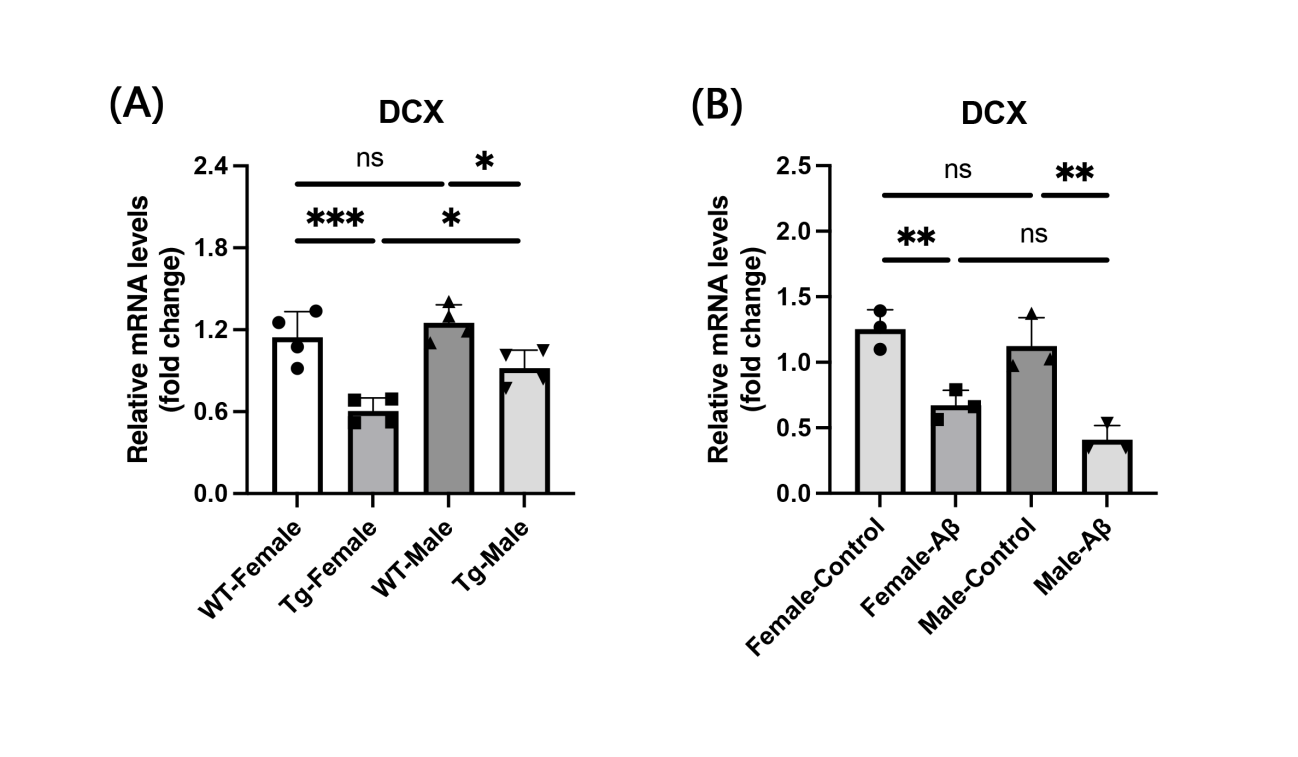


**Supplementary Figure** **1**. DCX expression in AD model mice. **A** and **B** The mRNA levels of DCX in APP^NL-G-F^ mice (**A**) and Aβ-injected mice (**B**) were measured by qRT-PCR. Data are presented as mean ± SEM; n = 3–4 per group. *p < 0.05; **p < 0.01; ***p < 0.001.


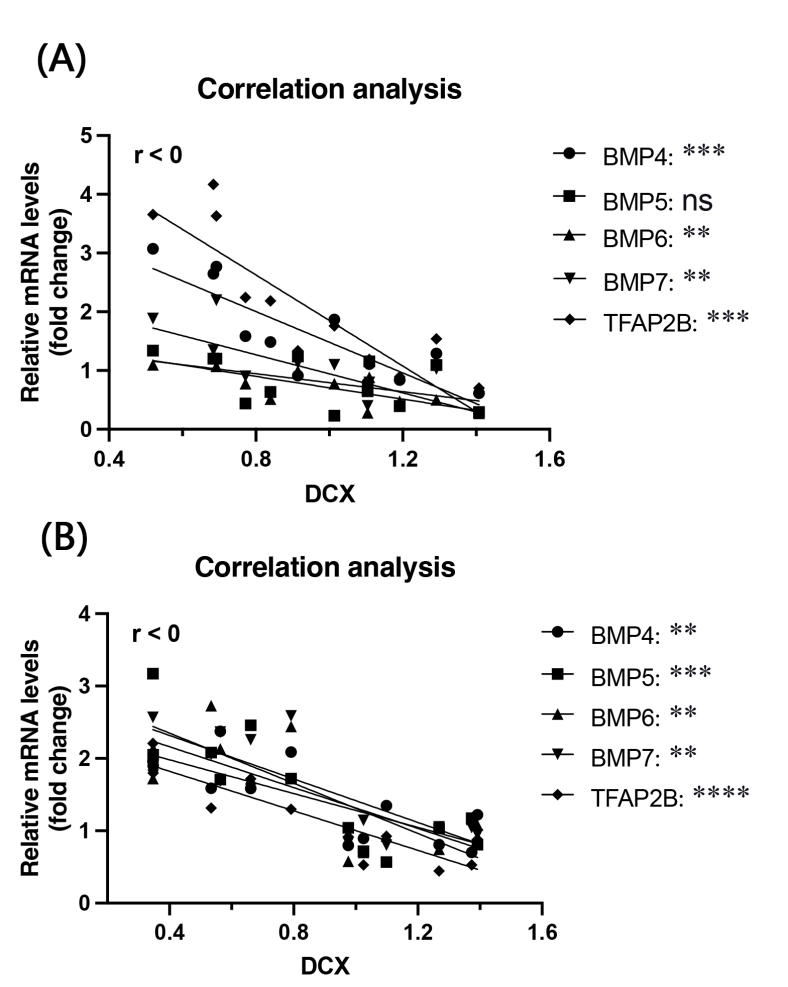


**Supplementary Figure** **2**. Correlation between the expressions of BMPs and TFAP2B with DCX. **A** and **B** Correlation analysis of BMPs and TFAP2B expression with DCX levels in APP^NL-G-F^ mice (**A**) and Aβ-injected mice (**B**). Pearson correlation analysis was performed; n = 12 per group. *p < 0.05; **p < 0.01; ***p < 0.001; ****p < 0.0001.
